# Temperature Trajectories Correlate With Cardiac Function in Patients With Sepsis

**DOI:** 10.1097/CCE.0000000000001282

**Published:** 2025-07-09

**Authors:** Annabel H. Lu, Vardhmaan Jain, Po-Han Chen, Matthew M. Churpek, Philip A. Verhoef, Arshed A. Quyyumi, Sivasubramanium V. Bhavani

**Affiliations:** 1 Emory University School of Medicine, Atlanta, GA.; 2 Department of Cardiovascular Medicine, Emory University School of Medicine, Atlanta, GA.; 3 Medical College of Georgia, Augusta, GA.; 4 Department of Medicine, University of Wisconsin, Madison, WI.; 5 Department of Medicine, University of Hawaii, Honolulu, HI.; 6 Division of Cardiology, Emory University School of Medicine, Atlanta, GA.; 7 Department of Medicine, Emory University, Atlanta, GA.

**Keywords:** group-based trajectory modeling, left ventricular dysfunction, right ventricular dysfunction, sepsis, temperature

## Abstract

**OBJECTIVES:**

Body temperature trajectories of infected patients are associated with dynamic clinical and immune responses to infection. Our objective was to evaluate the association between temperature trajectory subphenotypes and cardiac dysfunction determined by echocardiography.

**DESIGN:**

Retrospective cohort study.

**SETTING:**

Four hospitals within an academic healthcare system from 2016 to 2019.

**PATIENTS:**

Adult patients with suspicion of infection who underwent transthoracic echocardiography within 48 hours of admission.

**INTERVENTIONS:**

Using a validated model, patients were classified into four temperature trajectory subphenotypes. The primary outcome compared between subphenotypes was left ventricular dysfunction, defined as ejection fraction less than or equal to 50%.

**MEASUREMENTS AND MAIN RESULTS:**

One thousand nine hundred twenty-three hospitalized septic patients were classified into four subphenotypes: “hyperthermic, slow resolvers” (*n* = 264, 14%), “hyperthermic, fast resolvers” (302, 16%), “normothermic” patients (903, 47%), and “hypothermic” patients (454, 24%). Left ventricular and right ventricular dysfunction was significantly different between subphenotypes. Hypothermic patients exhibited the highest levels of left ventricular dysfunction (208, 46%; *p* < 0.01) and right ventricular dysfunction (169, 39%; *p* < 0.01). In the multivariable logistic regression analysis, adjusting for demographics, comorbidities, and severity of illness, membership in the hypothermic group (odds ratio, 2.65; 95% CI, 1.87–3.80; *p* < 0.01) was associated with significantly reduced left ventricular ejection fraction compared with hyperthermic slow resolvers as reference. Hypothermic patients also had the highest levels of vasopressor use (27%; *p* < 0.01), inotrope use (11%; *p* < 0.01), and in-hospital mortality (12%; *p* < 0.01).

**CONCLUSIONS:**

Temperature trajectories in sepsis are significantly associated with cardiac dysfunction, with hypothermic patients having the highest odds ratio of both left and right ventricular dysfunction. Bedside temperature monitoring could be a readily available marker to prompt early echocardiographic assessment, though further studies are needed to validate the relationship.

**KEY POINTS**

**Question:** Is there an association between temperature trajectory subphenotypes and cardiac dysfunction in patients with sepsis?

**Findings:** In this retrospective cohort study that included 1923 adults with suspicion of infection, significant differences were observed in left ventricular and right ventricular dysfunction between temperature trajectory subphenotypes. The hypothermic subphenotype had the highest rates of left ventricular and right ventricular dysfunction, even when adjusting for age and comorbidities.

**Meaning:** Temperature trajectory subphenotypes can serve as potential bedside indicators of cardiac dysfunction in patients with sepsis.

Sepsis, defined as life-threatening organ dysfunction in response to infection, is a leading cause of mortality and morbidity, representing approximately 20% of all global deaths (1). Cardiac dysfunction is seen in 20–60% of all septic patients and is associated with increased morbidity and mortality (2–4). Septic patients can manifest with left ventricular (LV), right ventricular (RV), and biventricular dysfunction, all of which portend worse short- and long-term outcomes (5, 6).

Septic patients also frequently present with temperature abnormalities, which have prognostic and clinical implications. Temperature regulation is a complex process that is affected by the cardiovascular system. Studies suggest that patients with heart failure have altered thermoregulation, leading to impaired temperature responses to stressors. In such populations, studies have demonstrated an association between temperature dysregulation and poor outcomes. In particular, hypothermia has been associated with LV dysfunction and increased mortality in heart failure populations (7–9). The host response to infection is a dynamic process with evolving physiologic responses. We have previously shown that longitudinal temperature trajectories can identify sepsis subphenotypes with distinct clinical characteristics and outcomes (10). Importantly, these subphenotypes correlate with dynamic clinical and immune responses to infection over the course of hospitalization (11). However, the relationship between longitudinal temperature response to infection and cardiac function has not been well characterized.

This study aims to bridge the knowledge gap between temperature responses and cardiac function in patients admitted with suspicion of infection. While the primary cohort includes all patients with suspected infection, the study also evaluates varyingly stringent definitions of infection, including sepsis (i.e., infection with organ dysfunction) to explore the relationship between temperature and cardiac function. We hypothesized that patients with cardiac dysfunction would be less likely to mount a febrile response to infection and would be more associated with a hypothermic temperature trajectory. The objectives of this current study are: 1) to classify a multicenter cohort of hospitalized patients with suspected infection into temperature trajectory subphenotypes and 2) to evaluate the association of these subphenotypes with cardiac dysfunction determined by echocardiography.

## METHODS

### Study Design and Data Collection

All adult patients with suspicion of infection admitted to four academic hospitals in the Emory Healthcare System between 2016 and 2019 were eligible for study inclusion in this retrospective cohort study. Patients were included if they underwent transthoracic echocardiography (TTE) within 48 hours of admission. Clinical characteristics, outcomes data, and *International Classification of Diseases*, 10th revision (ICD-10) codes at time of admission were retrospectively collected from hospital electronic medical records. The data were de-identified and made available on a secure server for analysis. This study was approved and the need for informed consent was waived by Emory University Institutional Review Board (IRB; STUDY00001815, “Clinical Trajectory Subphenotypes of Sepsis,” approved March 17, 2023) and was conducted in accordance with the ethical standards of Emory University IRB and the Helsinki Declaration of 1975.

### Criteria for Infection

Patients with suspicion of infection were identified by the following criteria: 1) having a blood culture order within 6 hours of admission and 2) receiving antibiotics within 6 hours of admission. Broad inclusion criteria for suspected infection were used because at the time of presentation patients may not yet meet the organ dysfunction criteria. The temperature trajectory model was developed to be generalizable to real world clinical practice, where they may be applied when infection is initially suspected but organ dysfunction is not yet confirmed (12).

### Exclusion Criteria

Patients were excluded if: 1) they were younger than 18 years old, 2) they did not meet above criteria for suspected infection, 3) they did not have documented oral temperature measurements, 4) they did not have a TTE obtained within 48 hours of admission, 5) ejection fraction was not documented on the patient’s TTE, and 6) they were not admitted through the Emergency Department.

### Temperature Trajectory Subphenotype Classification

Temperature measurements taken in the first 72 hours of hospitalization were included in the temperature trajectory algorithm. Only oral measurements were used for the analysis given that temperature measurements vary by site of measurement (e.g., oral, axillary, etc.). Oral temperatures were obtained with a Welch Allyn SureTemp Plus device (error rate, –0.016°C with monitor mode as reference). Patients were classified into one of four subphenotypes based on the previously validated model (10). The original model was derived from a cohort of 12,413 patients with all-cause infection and has been validated in multiple cohorts (including septic shock, COVID-19, and neutropenia) (11, 13, 14). The objective in developing the original model on a diverse, generalizable cohort was to then be able to apply it to specific cohorts, such as patients with varying cardiac function as in this present study. Four distinct temperature trajectory groups were identified: 1) “hyperthermic, slow resolvers”—patients with persistently elevated temperatures, 2) “hyperthermic, fast resolvers”—patients with elevated presenting temperature with defervescence over the ensuing 72 hours, 3) “normothermic”—patients with normal body temperatures, and 4) “hypothermic”—patients with low body temperatures. Each of the four temperature trajectories is a unique quadratic function expressing temperature as a function of time from presentation to the hospital (Temperature = β_0_ + β_1_ × Time + β2 × Time^2^). The temperature measurements from each patient were compared with the predicted measurement in each trajectory group. Patients were assigned to the group trajectory that resulted in the lowest squared residual error.

### Echocardiogram Data

Echocardiography was performed at the treating physician’s discretion. All TTE procedures were performed by registered diagnostic cardiac sonographers using commercially available ultrasound systems and read by experienced, board-certified cardiologists. Echocardiography data was obtained from electronic medical records and recorded in Research Electronic Data Capture. The following parameters were recorded for each echocardiogram: LV ejection fraction (LVEF) and RV systolic function. Patients were classified into two categories based on LVEF: low LVEF less than or equal to 50% and normal LVEF greater than 50%. LVEF was estimated visually according to standard clinical practice. When reported as a range, the minimum value was used to categorize LVEF. RV systolic function was assessed based on our institution’s standard TTE documentation, which reports RV systolic function as one of the following: “normal,” “indeterminate,” “mildly reduced,” “moderately reduced,” “severely reduced,” or as a range, for example, “moderate to severely reduced.” All echocardiogram readers at our institution comply with American Society of Echocardiography (15) standards of using both quantitative and qualitative parameters in evaluating RV function.

### Statistical Analysis

Categorical data was presented as *n* (%) and compared between subphenotypes using a chi-square test of independence. Quantitative data was summarized as median (interquartile range [IQR]) or mean (sd) and compared between subphenotypes using analysis of variance. Admission temperature and maximum temperature during hospitalization were compared between low and normal LVEF groups using a two-sample *t* test. Correlation between maximum temperature and LVEF as a continuous variable was assessed using Pearson correlation coefficient. Differences in echocardiographic parameters across temperature subphenotypes were analyzed using analysis of variance. These parameters included admission LVEF and RV systolic function.

### Multivariable Analysis of Primary and Secondary Outcomes

Multivariable logistic regression models were performed to evaluate the relationship between low LVEF and temperature trajectory, adjusting for variables that could have associations with both cardiac function and temperature. The logistic regression was adjusted based on the following explanatory variables: age, sex, race, ethnicity, history of coronary artery disease (CAD), chronic kidney disease (CKD), hypertension, diabetes mellitus (DM), use of vasopressors in the first 24 hours of admission, use of inotropes in the first 24 hours of admission, mechanical ventilation in the first 24 hours of admission, and maximum Sequential Organ Failure Assessment (SOFA) in the first 24 hours of admission. Vasopressors were defined as norepinephrine, epinephrine, vasopressin, angiotensin II, and phenylephrine. Inotropes were defined as dobutamine and milrinone. The subphenotype with the lowest rates of low LVEF was used as the reference group in order to highlight the differences between the extremes of the temperature trajectory subphenotypes. The analysis was also performed with the outcome of low LVEF defined using a cutoff value of LVEF less than or equal to 30%. The analysis was repeated for the outcome of RV dysfunction. Sensitivity analysis was performed in the subset of patients without an ICD-10 diagnosis of congestive heart failure (CHF) at admission.

#### Sensitivity Analysis of Infection Definitions

The primary analysis was repeated on patients with an ICD-10 diagnosis of sepsis and on patients with confirmed bacteremia.

#### Subgroup Analysis

The primary analysis was repeated on patients stratified by admission SOFA score using the following tertiles: SOFA less than 5, SOFA 5–7, and SOFA greater than 7.

### Trajectory of Cardiovascular Function

The following potential surrogates for trajectory of cardiovascular function were evaluated: percentage of patients on inotropes and vasopressors, pulse pressure (as a surrogate of cardiac output), and lactate and creatinine over time. In the subset of patients with repeat TTE performed within 1-year post-discharge, the association between subphenotype and LVEF was evaluated.

A *p* value of less than 0.05 were considered statistically significant. All statistical analyses were performed using R, Version 3.6.1 (R Foundation for Statistical Computing, Vienna, Austria).

## RESULTS

### Baseline Characteristics of Subphenotypes

Of 15,516 patients who were admitted to the hospital during the study period, 1,923 met the criteria for inclusion and were included in the final analysis (**eFig. 1**, https://links.lww.com/CCX/B522). The study population had a median age of 66 years (IQR, 53–77 yr), with 968 (50%) males, 20% receiving vasopressors, 5.6% receiving inotropes, and 25% receiving mechanical ventilation during the hospitalization (**Table 1**).

**TABLE 1.** Clinical Characteristics Compared Between Left Ventricular Ejection Fraction Groups

Characteristics	All Patients (*n* = 1923)	LVEF ≤ 50% (*n* = 662)	LVEF > 50% (*n* = 1261)	*p*
Age, yr, median (IQR)	66 (53–77)	67 (54–78)	65 (52–76)	< 0.01
Sex, male, *n* (%)	968 (50.3)	397 (60.0)	571 (45)	< 0.01
Admission temperature, °C, mean (sd)	37.0 (0.889)	36.8 (0.853)	37.1 (0.893)	< 0.01
Maximum temperature, °C, mean (sd)	37.4 (0.937)	37.3 (0.853)	37.5 (0.893)	< 0.01
Hispanic ethnicity, *n* (%)	58 (3.02)	21 (3.17)	37 (2.93)	0.88
Race, *n* (%)				
White	836 (43.5)	288 (43.5)	548 (43.5)	0.63
Black	981 (51.0)	342 (51.7)	639 (50.7)	
Other	106 (5.51)	32 (4.83)	74 (5.87)	
Comorbidities, *n* (%)				
Coronary artery disease	43 (2.24)	20 (3.02)	23 (1.82)	0.13
Chronic kidney disease	752 (39.1)	325 (49.1)	427 (33.9)	< 0.01
Hypertension	1508 (78.4)	549 (82.9)	959 (76.1)	< 0.01
Diabetes mellitus	735 (38.2)	273 (41.2)	462 (36.6)	0.054
Hospital outcomes				
Vasopressors, *n* (%)	384 (20.0)	166 (25.1)	218 (17.3)	< 0.01
Inotropes, *n* (%)	108 (5.62)	96 (14.5)	12 (0.952)	< 0.01
Mechanical ventilation, *n* (%)	475 (24.7)	217 (32.8)	258 (20.5)	< 0.01
Sequential Organ Failure Assessment, median (IQR)	5 (4–7)	6 (4–8)	5 (4–7)	< 0.01
In-hospital mortality, *n* (%)	129 (6.71)	59 (8.91)	70 (5.55)	< 0.01

IQR = interquartile range, LVEF = left ventricular ejection fraction.

Vasopressors were defined as norepinephrine, epinephrine, vasopressin, angiotensin II, and phenylephrine. Inotropes were defined as dobutamine and milrinone. *p* values signify the results of comparisons between subphenotypes through χ^2^ test or analysis of variance testing, as appropriate.

Patients were classified into one of four validated temperature subphenotypes: “hyperthermic, slow resolvers” (*n* = 264, 14%), “hyperthermic, fast resolvers” (302, 16%), “normothermic” patients (903, 47%), and “hypothermic” patients (454, 24%) (**eFig. 2**, https://links.lww.com/CCX/B522). Age was significantly different between subphenotypes (*p* < 0.01): hyperthermic slow resolvers were the youngest with a median age of 60 years (46–71 yr), whereas hypothermic patients were the oldest with a median age of 68 years (56–78 yr). Several comorbidities were significantly different, with hypothermic patients having the highest prevalence of CKD (46%; *p* < 0.01) and hypertension (83%; *p* < 0.01). Hypothermic patients had the highest rates of vasopressor (27%; *p* < 0.01) and inotrope use (11%; *p* < 0.01). The overall in-hospital mortality rate was significantly different between subphenotypes (*p* < 0.01): 11.7% in hypothermic patients, 6.4% in hyperthermic slow resolvers, 5.0% in hyperthermic fast resolvers, and 4.9% in normothermic patients (**eTable 1**, https://links.lww.com/CCX/B522).

### Temperature and LVEF

**Figure 1.**
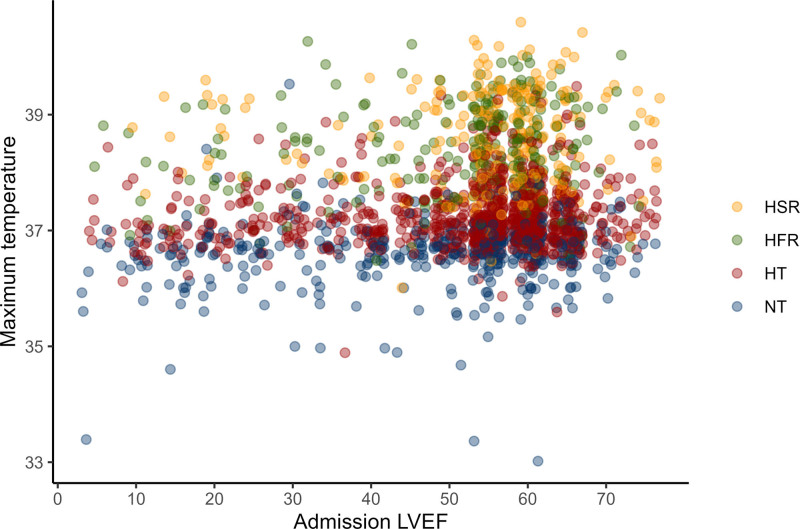
Relationship between left ventricular ejection fraction (LVEF) and maximum temperature. Presented are the LVEF and maximum temperature. The *points* are *colored* as per the subphenotype. *Points* are jittered by 2% ejection fraction so that the individual points are better visualized. There is a positive linear relationship between LVEF and presenting maximum temperature. HFR = hyperthermic, fast resolvers, HSR = hyperthermic, slow resolvers, HT = hypothermic, NT = normothermic.

The mean LVEF for the study cohort was 51% (sd, 15.3). A total of 662 patients (34%) had low LVEF, with a mean of 34% (14). The low LVEF group had a lower admission temperature at 36.8°C (sd, 0.85) compared with the normal LVEF group at 37.1°C (0.89). The low LVEF group had a lower maximum temperature during hospitalization at 37.3°C (0.85) compared with the normal LVEF group at 37.5°C (0.89). Maximum temperature had a significant positive correlation with LVEF (*r* = +0.15; *p* < 0.01; **Fig. 1**).

### Association of Subphenotypes With LVEF Less Than or Equal to 50%

**Figure 2.**
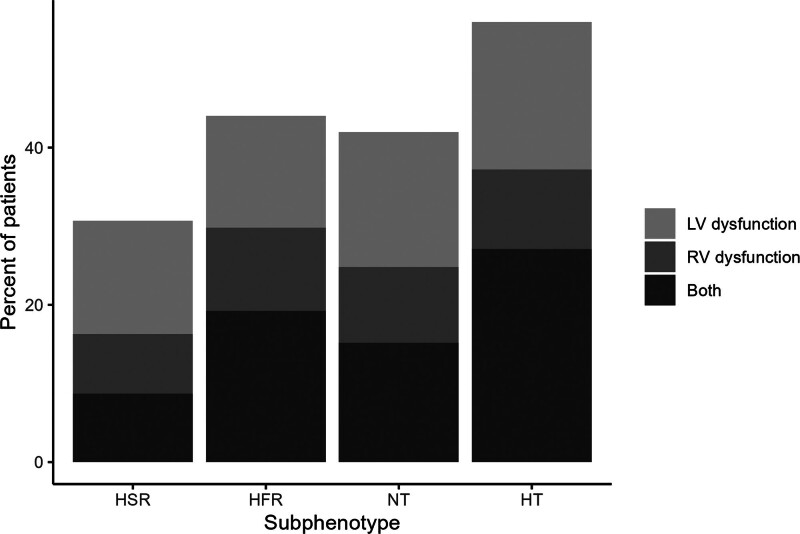
Echocardiographic parameters compared between temperature subphenotypes. Echocardiographic parameters were compared between the temperature trajectory subphenotypes using the χ^2^ test. The *x*-axis presents the four temperature subphenotypes. The *y*-axis represents the percentage of patients within each subphenotype cohort. Hypothermic (HT) patients had the highest levels of left ventricular (LV) dysfunction (208, 46%; *p* < 0.01) and right ventricular (RV) systolic dysfunction (169, 39%; *p* < 0.01) compared with hyperthermic slow resolvers (HSRs; 61, 23% and 43, 17%, respectively). HFR = hyperthermic, fast resolvers, NT = normothermic.

The mean LVEF was significantly different between subphenotypes (*p* < 0.01), with hyperthermic slow resolvers having a mean LVEF of 54.6% (12.5) and hypothermic patients having a mean LVEF of 47.1% (17.3) (**eFig. 3**, https://links.lww.com/CCX/B522). Hypothermic patients had the highest prevalence of low LVEF (208, 46%), followed by hyperthermic fast resolvers (101, 33%), normothermic patients (292, 32%), and hyperthermic slow resolvers (61, 23%) (**Fig. 2**; and **eTable 2**, https://links.lww.com/CCX/B522).

On logistic regression, when adjusting for age, sex, race, ethnicity, comorbidities (CAD, CKD, hypertension, DM), and SOFA score, with hyperthermic slow resolvers as reference, hypothermic patients had significantly increased odds ratio (OR) of low LVEF (OR, 2.65; 95% CI, 1.87–3.80; *p* < 0.01) (**eTable 3**, https://links.lww.com/CCX/B522).

We performed a sensitivity analysis using normothermic patients as reference. The hypothermic subphenotype remained significantly associated with higher odds of reduced cardiac function (OR, 1.58; 95% CI, 1.24–2.01; *p* < 0.01), while hyperthermic slow resolvers were associated with lower odds of reduced cardiac function (OR, 0.60; 95% CI, 0.43–0.83; *p* < 0.01) (**eFig. 4**, https://links.lww.com/CCX/B522).

### Sensitivity Analysis of Association of Subphenotypes With LVEF Less Than or Equal to 30%

A sensitivity analysis was performed using a cutoff value of LVEF less than or equal to 30% as low LVEF. Two hundred seventy-six patients had LVEF less than or equal to 30% (21 hyperthermic slow resolvers, 41 hyperthermic fast resolvers, 116 normothermic patients, and 98 hypothermic patients). We found that hypothermic patients had a significantly increased OR of LVEF less than or equal to 30% (OR, 3.26; 95% CI, 1.95–5.44; *p* < 0.01) (**eFig. 5** and **eTable 4**, https://links.lww.com/CCX/B522).

To investigate the association without cutoffs, we performed a linear regression evaluating the association of subphenotype with LVEF as a continuous variable and found that adjusting for confounders, hypothermic patients were associated with 7% lower ejection fraction compared with hyperthermic slow resolvers as reference (–7.0; 95% CI, –4.8 to –9.3; *p* < 0.01).

### Association of Subphenotypes With Right Ventricular Systolic Function

RV systolic function was significantly different between subphenotypes (*p* < 0.01). Hypothermic patients had the highest rate of RV systolic dysfunction (169, 39%), followed by hyperthermic fast resolvers (90, 31%), normothermic patients (224, 26%), and hyperthermic slow resolvers (43, 17%) (Fig. 2; and eTable 2, https://links.lww.com/CCX/B522).

### Sensitivity Analyses on Varying Inclusion Criteria

**Figure 3.**
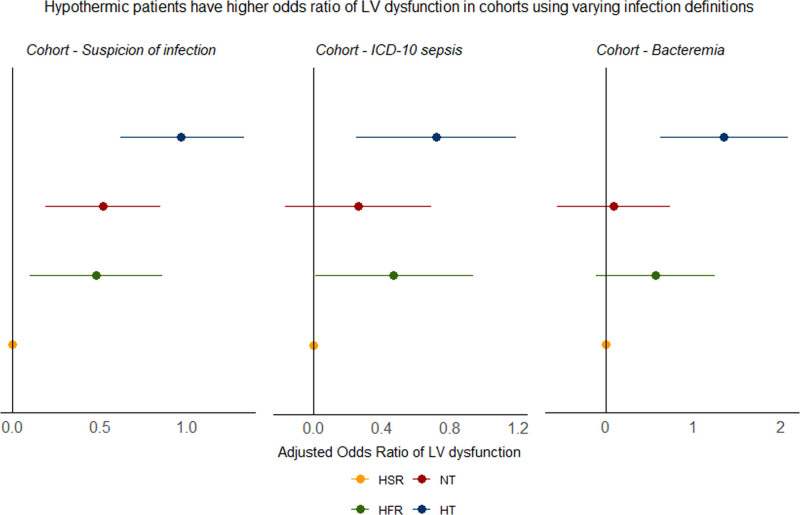
Adjusted odds ratio for left ventricular (LV) dysfunction across cohorts. We present the adjusted odds ratio of the subphenotypes for low LV ejection fraction (LVEF) in three cohorts: 1) initial cohort of patients with suspicion of infection, 2) patients with *International Classification of Diseases*, 10th revision (ICD-10) diagnosis of sepsis, and 3) patients with confirmed bacteremia. Hyperthermic slow resolvers (HSRs) served as the reference group. Hypothermic (HT) patients had increased odds ratio of low LVEF in all three cohorts. HFR = hyperthermic, fast resolvers, NT = normothermic.

A sensitivity analysis was performed in a subset of 1841 patients with no prior ICD-10 diagnosis of CHF at time of admission. Hypothermic patients had significantly higher OR of low LVEF (OR, 2.52; 95% CI, 1.76–3.65; *p* < 0.01) (**eTable 5**, https://links.lww.com/CCX/B522). A sensitivity analysis was performed on a subset of 893 patients with an ICD-10 diagnosis of sepsis, which demonstrated similar results, with hypothermic patients having a significantly higher OR of low LVEF (OR, 2.06; 95% CI, 1.28–3.3; *p* < 0.01) (**eTable 6**, https://links.lww.com/CCX/B522). We also performed a sensitivity analysis on a subset of 431 patients with confirmed bacteremia. Among this cohort of bacteremic patients, hypothermic patients had a significantly higher OR of low LVEF (OR, 3.85; 95% CI, 1.86–7.99; *p* < 0.01). These results are similar to our initial cohort analysis (**Fig. 3**; and **eTable 7**, https://links.lww.com/CCX/B522).

### Stratified Analysis by SOFA Score

When adjusting for SOFA score at admission, the hypothermic group remained significantly associated with low LVEF (OR, 2.65; 95% CI, 1.87–3.90; *p* < 0.01). We also present stratified analyses by SOFA tertile and found that across SOFA tertiles, hypothermic patients had a significant association with low LVEF (**eFig. 6** and **eTable 8**, https://links.lww.com/CCX/B522).

### Trajectory of Cardiac Function

**Figure 4.**
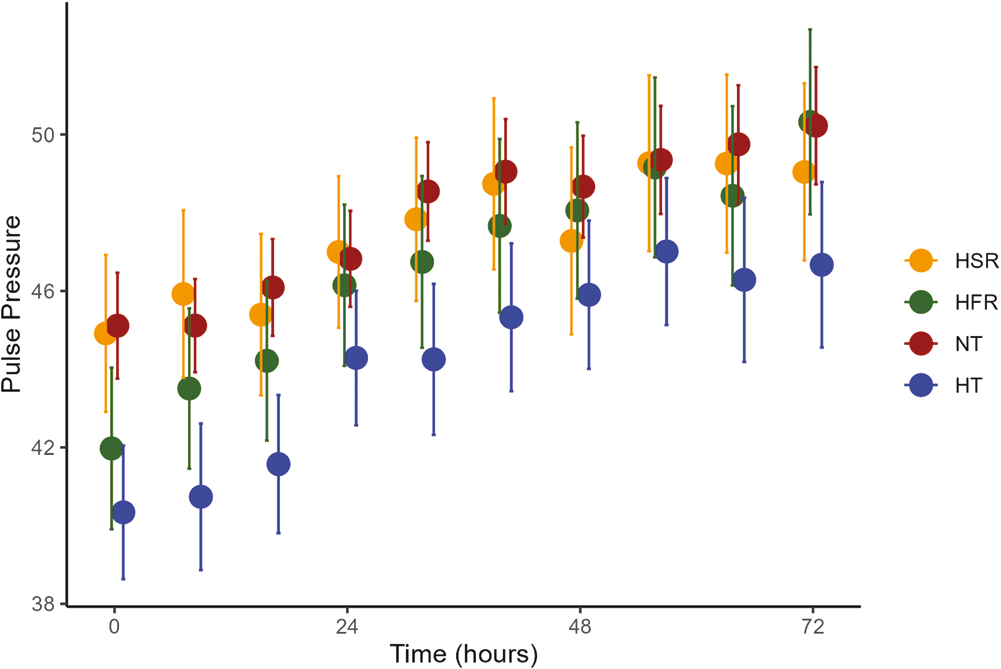
Pulse pressure compared between temperature trajectory subphenotypes. Hypothermic (HT) patients had the narrowest pulse pressure compared with the other subphenotypes, and this persisted over the first 72 hr of hospitalization. HFR = hyperthermic, fast resolvers, HSR = hyperthermic, slow resolvers, NT = normothermic.

We evaluated the following surrogates for trajectory of cardiovascular function: percentage of patients on inotropes and vasopressors, pulse pressure, and lactate and creatinine over time. The hypothermic subphenotype had the highest percentage of patients receiving inotropes, with over three times higher rates of use compared with the hyperthermic subphenotypes. Hypothermic patients also had the highest percentage of patients receiving vasopressors, with over 20% of hypothermic patients receiving vasopressors in the first day of hospitalization (**eFig. 7**, https://links.lww.com/CCX/B522). Hypothermic patients had the narrowest pulse pressure compared with the other subphenotypes (**Fig. 4**). Hypothermic patients had higher lactate and creatinine levels (**eFig. 8**, https://links.lww.com/CCX/B522).

Finally, we evaluated a subset of patients with repeat TTEs in the 1-year period post-discharge. Five hundred twenty-seven patients had repeat TTE’s, with 71 hyperthermic slow resolvers (representing 27% of hyperthermic slow resolvers in the original study cohort), 86 hyperthermic fast resolvers (28%), 227 normothermic patients (25%), and 143 hypothermic patients (31%). On the post-discharge TTE, the association between the hypothermic subphenotype and low LVEF persisted (OR, 3.29; 95% CI, 1.71–6.32; *p* < 0.01) (**eFig. 9** and **eTable 9**, https://links.lww.com/CCX/B522).

## DISCUSSION

To our knowledge, this is the first study to demonstrate a significant association between static and dynamic temperature measurements and cardiac function in patients with sepsis. We found that the hypothermic subphenotype had the highest rates of LV and RV dysfunction, even when adjusting for age, comorbidities, and severity of illness. Hypothermic patients were significantly more likely to receive vasopressors and inotropes and had the highest mortality.

The relationship between body temperature and cardiovascular function is complex and bidirectional. While cardiac function likely affects the temperature response to an infection, the temperature response itself may also affect cardiac function. For instance, elevated temperatures could lead to increased cardiac output and vasodilation, with recent studies suggesting a beneficial physiologic effect of external warming in septic patients (16–18). However, the metabolic demands of mounting a fever can itself increase workload, which may adversely affect patients with cardiac dysfunction (19). Patients with cardiac dysfunction may also have alterations in cardiac output and vascular tone, which could affect heat generation and dissipation (20) and thus their temperature response to an infection. Additionally, patients with cardiac dysfunction have excess activation of the renin-angiotensin system, which may result in reduced thermogenic capacity and an impaired febrile response. Angiotensin II has been implicated in both physiologic and pathologic thermoregulation (21), with animal studies suggesting that central administration of angiotensin II reduces body temperature through decreased metabolic heat production and increased heat dissipation (22, 23). In humans, increased renin-angiotensin system activation in advanced cardiac disease has been hypothesized to contribute to hypothermia in heart failure (9). It is unclear whether this mechanism could affect temperature responses in patients with mild and moderately reduced cardiac function.

Previous literature has demonstrated that heart failure patients presenting with hypothermia have increased mortality (7–9). Consistent with these studies, when examining a population of septic patients, our study found that hypothermic patients had the highest mortality among the temperature subphenotypes. When limited to patients without a prior ICD-10 diagnosis of CHF, low LVEF remained significantly different between subphenotypes with highest rates in hypothermic patients. Further investigation is needed to determine the direction of causality in these results and distinguish between patients with preexisting and new onset cardiac dysfunction. The cornerstone of management of cardiac dysfunction in sepsis is early identification, namely by echocardiography, and initiation of timely therapy. The use of TTE in septic patients has been associated with improved 28-day mortality, likely in part due to the initiation of therapies as indicated and guided by TTE results (24). The universally available bedside measurement of temperature can identify patients who may benefit from echocardiography, particularly in resource-limited settings. Additionally, it could aid clinicians in the precision management of patients, including earlier initiation of inotropes, more conservative fluid resuscitation, and early diuresis (25). Indeed, our study found that hypothermic patients were two to three times more likely to receive inotropes during their hospitalization compared with other temperature trajectories. Further investigation is required to evaluate whether temperature trajectory can serve as a clinical marker in the identification of cardiogenic shock, and whether hypothermic patients benefit from therapies targeting this pathophysiology.

RV dysfunction is frequently observed in patients with sepsis. One study by Hiraiwa et al (26) found that visually assessed RV dysfunction in septic shock patients was associated with increased mortality, fatal arrhythmias, and circulatory insufficiency. Interventions that increase RV afterload and preload such as positive end-expiratory pressure and fluid administration can be detrimental in these patients (27). In our study, more than one in three hypothermic patients had RV dysfunction, which in part could be contributing to the increased mortality in this cohort. Early identification of patients at risk for RV dysfunction can allow clinicians to tailor acute and long-term management strategies.

Our study had several limitations. First, the retrospective nature of the study limits causal inferences. Specifically, it is difficult to disentangle the directionality of influence between temperature and cardiac function. Second, echocardiographic measurements of LVEF and RV function are affected by preload and afterload. Thus, receipt of interventions can lead to under-recognition of LV and RV dysfunction. Third, there is no consensus threshold for low LVEF and it differs among guidelines, further raising potential for misclassification of patients into LVEF groups. RV function assessment on TTE is based on both qualitative and quantitative measures, and as with any imaging modality, the possibility of interobserver variation exists. Fourth, clinical data including TTE’s were ordered as part of routine care. The exclusion of patients without TTEs may predispose patient selection to those with preexisting cardiac disease, concern for shock, or other factors that might impact temperature assessment. Fifth, we did not have baseline TTE data for patients prior to their admission, so we could not definitively identify preexisting vs. new onset cardiac dysfunction. To account for this, we used the ICD-10 diagnosis of CHF to conduct a sensitivity analysis limited to patients without CHF; however, this is not a perfect surrogate, and there is the possibility of error due to misclassification. Finally, hypothermic patients had higher creatinine levels, lactate levels, SOFA scores, and overall had higher severity of illness. While the higher severity of illness may confound the association between subphenotype and cardiac dysfunction, the association remained significant even under varying tertiles of SOFA scores.

## CONCLUSIONS

We present the novel finding of the association between longitudinal temperature trajectory and cardiac function in septic patients. Bedside temperature monitoring could be a readily available marker to prompt early echocardiographic assessment, although further studies are needed to validate the relationship.

## Supplementary Material
