# Supplementary material for: Temperature Trajectories Correlate With Cardiac Function in Patients With Sepsis
Source: Crit Care Explor. 2025 Jul 9;7(7):e1282. doi: 10.1097/CCE.0000000000001282 (PMC12440545; doi:10.1097/CCE.0000000000001282)

SUPPLEMENTAL DIGITAL CONTENT

Table of Contents

1. eTable 1. Clinical Characteristics Compared Between Temperature Trajectory Subphenotypes
2. eTable 2. Echocardiographic Parameters Compared Between Temperature Subphenotypes
3. eTable 3. Logistic Regression for Left Ventricular Ejection Fraction ≤ 50%
4. eTable 4. Logistic Regression for Left Ventricular Ejection Fraction ≤ 30%
5. eTable 5. Logistic Regression for Left Ventricular Ejection Fraction ≤ 50% in Patients Without Prior ICD-10 Diagnosis of CHF
6. eTable 6. Logistic Regression for Left Ventricular Ejection Fraction ≤ 50% in Patients With ICD-10 Diagnosis of Sepsis
7. eTable 7. Logistic Regression for Left Ventricular Ejection Fraction ≤ 50% in Patients With Confirmed Bacteremia
8. eTable 8. Stratified Analysis of Left Ventricular Ejection Fraction ≤ 50% by SOFA score
9. eTable 9. Logistic Regression for 1-year Post Discharge Left Ventricular Ejection Fraction ≤ 50%
10. eFigure 1. Consolidated Standards of Reporting Trials diagram of patient selection for the study population.
11. eFigure 2. Temperature trajectory subphenotypes in patients with suspected infection.
12. eFigure 3. Mean Admission Left Ventricular Ejection Fraction by Subphenotype.
13. eFigure 4. Sensitivity Analysis with Normothermics as reference group.
14. eFigure 5. Odds Ratio for LVEF ≤ 30% compared between temperature trajectory subphenotypes.
15. eFigure 6. Stratified Analysis by SOFA score.
16. eFigure 7. Inotropes and Vasopressors compared between temperature trajectory subphenotypes.
17. eFigure 8. Biomarker levels over time compared between temperature trajectory subphenotypes.
18. eFigure 9. 1-year Post Discharge LV dysfunction compared between temperature trajectory subphenotypes.

eTable 1. Clinical Characteristics Compared Between Temperature Trajectory Subphenotypes

Characteristics	Hyperthermic Slow Resolvers (n=264)	Hyperthermic Fast Resolvers (n=302)	Normothermic (n=903)	Hypothermic (n=454)	*P value*
Age, yr, *n* (%)	60 (46-71)	63 (47-74)	67 (54-78)	68 (56-78)	*<0.01*
Sex, male, *n* (%)	138 (52.3)	157 (52.0)	442 (48.9)	231 (50.9)	*0.69*
Admission temperature, °C, mean (SD)	37.8 (0.926)	37.9 (0.886)	36.8 (0.515)	36.3 (0.620)	*<0.01*
Maximum temperature, °C, mean (SD)	38.6 (0.926)	38.3 (0.886)	37.2 (0.515)	36.6 (0.620)	*<0.01*
Hispanic ethnicity, *n* (%)	11 (4.17)	5 (1.66)	30 (3.32)	12 (2.64)	*0.31*
Race					
White, *n* (%)	97 (36.7)	121 (40.1)	426 (47.2)	192 (42.3)	*<0.01*
Black, *n* (%)	150 (56.8)	167 (55.3)	420 (46.5)	244 (53.7)	
Other, *n* (%)	17 (6.44)	14 (4.64)	57 (6.31)	18 (3.96)	
Comorbidities					
CAD, *n* (%)	5 (1.89)	6 (1.99)	21 (2.33)	11 (2.42)	*0.95*
CKD, *n* (%)	96 (36.4)	121 (40.1)	325 (36.0)	210 (46.3)	*<0.01*
Hypertension, *n* (%)	189 (71.6)	220 (72.8)	721 (79.8)	378 (83.3)	*<0.01*
DM, *n* (%)	88 (33.3)	108 (35.8)	349 (38.6)	190 (41.9)	*0.11*
Hospital outcomes					
Vasopressors, *n* (%)	48 (18.2)	66 (21.9)	147 (16.3)	123 (27.1)	*<0.01*
Inotropes, *n* (%)	10 (3.79)	10 (3.31)	40 (4.43)	48 (10.6)	*<0.01*
Mechanical ventilation, *n* (%)	83 (31.4)	67 (22.2)	187 (20.7)	138 (30.4)	*<0.01*

SOFA, median (IQR)	5 (4-7)	6 (4-8)	5 (3-7)	6 (4-8)	*0.26*
In-hospital mortality, *n* (%)	17 (6.44)	15 (4.97)	44 (4.87)	53 (11.7)	*<0.01*

Vasopressors were defined as norepinephrine, epinephrine, vasopressin, angiotensin II, and phenylephrine. Inotropes were defined as dobutamine and milrinone. P-values signify the results of comparisons between subphenotypes through chi-square test or analysis of variance testing, as appropriate. Definition of abbreviations: CAD = coronary artery disease; CKD = chronic kidney disease; DM = diabetes mellitus; SOFA = Sepsis-related Organ Failure Assessment.

eTable 2. Echocardiographic Parameters Compared Between Temperature Subphenotypes

**Echocardiographic Parameter**	**All Patients (n=1,923)**	**Hyperthermic Slow Resolvers (n=264)**	**Hyperthermic Fast Resolvers (n=302)**	**Normothermic (n=903)**	**Hypothermic (n=454)**	***P value***
LVEF						*<0.01*
LVEF > 50%, *n* (%)	1,261 (65.6)	203 (76.9)	201 (66.6)	611 (67.7)	246 (54.2)	
LVEF ≤ 50%, *n* (%)	662 (34.4)	61 (23.1)	101 (33.4)	292 (32.3)	208 (45.8)	
RV Systolic Function						*<0.01*
Normal, *n* (%)	1,302 (71.2)	203 (82.5)	199 (68.9)	639 (74.0)	261 (60.7)	
Abnormal, *n* (%)	526 (28.8)	43 (17.5)	90 (31.1)	224 (26.0)	169 (39.3)	

All values are presented as n (%). P values signify the results of comparisons between subphenotypes through chi-square tests.

eTable 3. Logistic Regression for Left Ventricular Ejection Fraction ≤ 50%

**Variable**	**OR**	**95% CI**	***P value***
Age	1.00	1.00-1.01	*0.28*
Sex	0.55	0.45-0.67	*<0.01*
SOFA	1.06	1.02-1.10	*<0.01*
Hispanic ethnicity	1.33	0.73-2.37	*0.35*
Race			
White	1.00	-	-
Black	0.98	0.79-1.22	*0.88*
Other	0.91	0.57-1.44	*0.69*
Comorbidities			
CAD	1.52	0.80-2.86	*0.19*
CKD	1.59	1.28-1.98	*<0.01*
Hypertension	1.09	0.83-1.44	*0.55*
DM	1.00	0.80-1.22	*0.90*
Temperature Subphenotype			
Hyperthermic slow resolvers	1.00	-	-
Hyperthermic fast resolvers	1.62	1.10-2.38	*<0.05*
Normothermic	1.68	1.21-2.35	*<0.01*
Hypothermic	2.65	1.87-3.80	*<0.01*

Definition of abbreviations: OR = odds ratio; CI = confidence interval; CAD = coronary artery disease; CKD = chronic kidney disease; DM = diabetes mellitus; SOFA = Sepsis-related Organ Failure Assessment

eTable 4. Logistic Regression for Left Ventricular Ejection Fraction ≤ 30%

**Variable**	**OR**	**95% CI**	***P value***
Age	0.99	0.98-1.00	0.023
Sex	0.54	0.41-0.72	*<0.01*
SOFA	1.07	1.02-1.12	*<0.01*
Hispanic ethnicity	1.52	0.71-3.21	0.28
Race			
White	1.00	-	-
Black	1.14	0.85-1.54	0.38
Other	1.02	0.53-1.95	0.96
Comorbidities			
CAD	1.55	0.71-3.39	0.27
CKD	1.63	1.21-2.19	*<0.01*
Hypertension	1.39	0.92-2.08	0.12
DM	1.09	0.82-1.44	0.55
Temperature Subphenotype			
Hyperthermic slow resolvers	0.07	0.03-0.16	*<0.01*
Hyperthermic fast resolvers	1.79	1.01-3.14	0.045
Normothermic	1.96	1.19-3.23	*<0.01*
Hypothermic	3.26	1.95-5.44	*<0.01*

Definition of abbreviations: OR = odds ratio; CI = confidence interval; CAD = coronary artery disease; CHF = congestive heart failure; CKD = chronic kidney disease; DM = diabetes mellitus; SOFA = Sepsis-related Organ Failure Assessment

eTable 5. Logistic Regression for Left Ventricular Ejection Fraction ≤ 50% in Patients Without Prior ICD-10 Diagnosis of CHF

**Variable**	**OR**	**95% CI**	***P value***
Age	1.01	1.00-1.01	*0.12*
Sex	0.54	0.44-0.66	*<0.01*
SOFA	1.06	1.02-1.09	*<0.01*
Hispanic ethnicity	1.41	0.77-2.53	*0.25*
Race			
White	1.00	-	-
Black	0.96	0.76-1.20	*0.70*
Other	0.91	0.56-1.45	*0.71*
Comorbidities			
CAD	1.30	0.65-2.52	*0.45*
CKD	1.62	1.29-2.03	*<0.01*
Hypertension	1.07	0.81-1.43	*0.63*
DM	0.96	0.77-1.20	*0.74*
Temperature Subphenotype			
Hyperthermic slow resolvers	1.00	-	-
Hyperthermic fast resolvers	1.52	1.03-2.27	*<0.05*
Normothermic	1.61	1.15-2.27	*<0.01*
Hypothermic	2.52	1.76-3.65	*<0.01*

Definition of abbreviations: OR = odds ratio; CI = confidence interval; CAD = coronary artery disease; CHF = congestive heart failure; CKD = chronic kidney disease; DM = diabetes mellitus; SOFA = Sepsis-related Organ Failure Assessment

eTable 6. Logistic Regression for Left Ventricular Ejection Fraction ≤ 50% in Patients With ICD-10 Diagnosis of Sepsis

**Variable**	**OR**	**95% CI**	***P value***
Age	1.01	1.00-1.02	*0.15*
Sex	0.60	0.45-0.81	*<0.01*
SOFA	1.07	1.02-1.12	*<0.01*
Hispanic ethnicity	1.09	0.45-2.49	*0.84*
Race			
White	1.00	-	-
Black	1.06	0.77-1.47	*0.71*
Other	1.07	0.53-2.10	*0.85*
Comorbidities			
CAD	1.66	0.57-5.02	*0.35*
CKD	1.51	1.10-2.07	*<0.05*
Hypertension	1.14	0.77-1.70	*0.52*
DM	0.87	0.63-1.18	*0.37*
Temperature Subphenotype			
Hyperthermic slow resolvers	1.00	-	-
Hyperthermic fast resolvers	1.61	1.01-2.57	*0.05*
Normothermic	1.29	0.85-2.00	*0.24*
Hypothermic	2.06	1.28-3.32	*<0.01*

Definition of abbreviations: OR = odds ratio; CI = confidence interval; CAD = coronary artery disease; CKD = chronic kidney disease; DM = diabetes mellitus; SOFA = Sepsis-related Organ Failure Assessment

eTable 7. Logistic Regression for Left Ventricular Ejection Fraction ≤ 50% in Patients With Confirmed Bacteremia

Variable	OR	95% CI	*P value*
Age	1.00	0.99-1.02	0.68
Sex	0.49	0.32-0.76	*<0.01*
SOFA	1.04	0.97-1.12	0.22
Hispanic ethnicity	2.72	0.86-8.59	0.088
Race			
White	1.00	-	-
Black	0.98	0.62-1.57	0.94
Other	0.42	0.14-1.33	0.14
Comorbidities			
CAD	3.89	0.97-15.66	0.056
CKD	1.39	0.87-2.22	0.17
Hypertension	1.29	0.72-2.30	0.39
DM	0.72	0.45-1.15	0.17
Temperature Subphenotype			
Hyperthermic slow resolvers	0.26	0.08-0.81	0.02
Hyperthermic fast resolvers	1.77	0.90-3.48	0.099
Normothermic	1.09	0.58-2.08	0.78
Hypothermic	3.85	1.86-7.99	*<0.01*

Definition of abbreviations: OR = odds ratio; CI = confidence interval; CAD = coronary artery disease; CKD = chronic kidney disease; DM = diabetes mellitus; SOFA = Sepsis-related Organ Failure Assessment

eTable 8. Stratified Analysis of Left Ventricular Ejection Fraction ≤ 50% by SOFA score

SOFA score	Temperature Subphenotype	OR	95% CI	*P value*
<5	Hyperthermic slow resolvers	0.18	0.06-0.48	*<0.01*
	Hyperthermic fast resolvers	1.82	0.81-4.08	0.15
	Normothermic	2.03	1.05-3.93	0.035
	Hypothermic	3.08	1.51-6.30	*<0.01*
5-7	Hyperthermic slow resolvers	0.02	0.00-0.09	*<0.01*
	Hyperthermic fast resolvers	2.18	1.08-4.40	0.03
	Normothermic	2.31	1.26-4.24	*<0.01*
	Hypothermic	3.67	1.89-7.13	*<0.01*
>7	Hyperthermic slow resolvers	0.69	0.22-2.19	0.53
	Hyperthermic fast resolvers	1.11	0.62-1.98	0.74
	Normothermic	1.09	0.64-1.85	0.74
	Hypothermic	1.84	1.07-3.17	0.028

Definition of abbreviations: OR = odds ratio; CI = confidence interval; CAD = coronary artery disease; CKD = chronic kidney disease; DM = diabetes mellitus; SOFA = Sepsis-related Organ Failure Assessment

eTable 9. Logistic Regression for 1-year Post Discharge Left Ventricular Ejection Fraction ≤ 50%

**Variable**	**OR**	**95% CI**	***P value***
Age	0.99	0.98-1.01	0.39
Sex	0.55	0.38-0.80	*<0.01*
SOFA	1.02	0.95-1.09	0.58
Hispanic ethnicity	1.77	0.50-6.29	0.38
Race			
White			
Black	1.08	0.71-1.64	0.71
Other	1.21	0.48-3.08	0.69
Comorbidities			
CAD	1.03	0.37-2.91	0.95
CKD	1.1	0.73-1.65	0.64
Hypertension	1.99	1.09-3.64	0.025
DM	1.37	0.94-2.01	0.1
Temperature Subphenotype			
Hyperthermic slow resolvers	0.25	0.09-0.73	0.011
Hyperthermic fast resolvers	1.51	0.74-3.10	0.26
Normothermic	2.08	1.12-3.86	0.021
Hypothermic	3.29	1.71-6.32	*<0.01*

Definition of abbreviations: OR = odds ratio; CI = confidence interval; CAD = coronary artery disease; CKD = chronic kidney disease; DM = diabetes mellitus; SOFA = Sepsis-related Organ Failure Assessment

eFigure 1. Consolidated Standards of Reporting Trials diagram of patient selection for the study population.

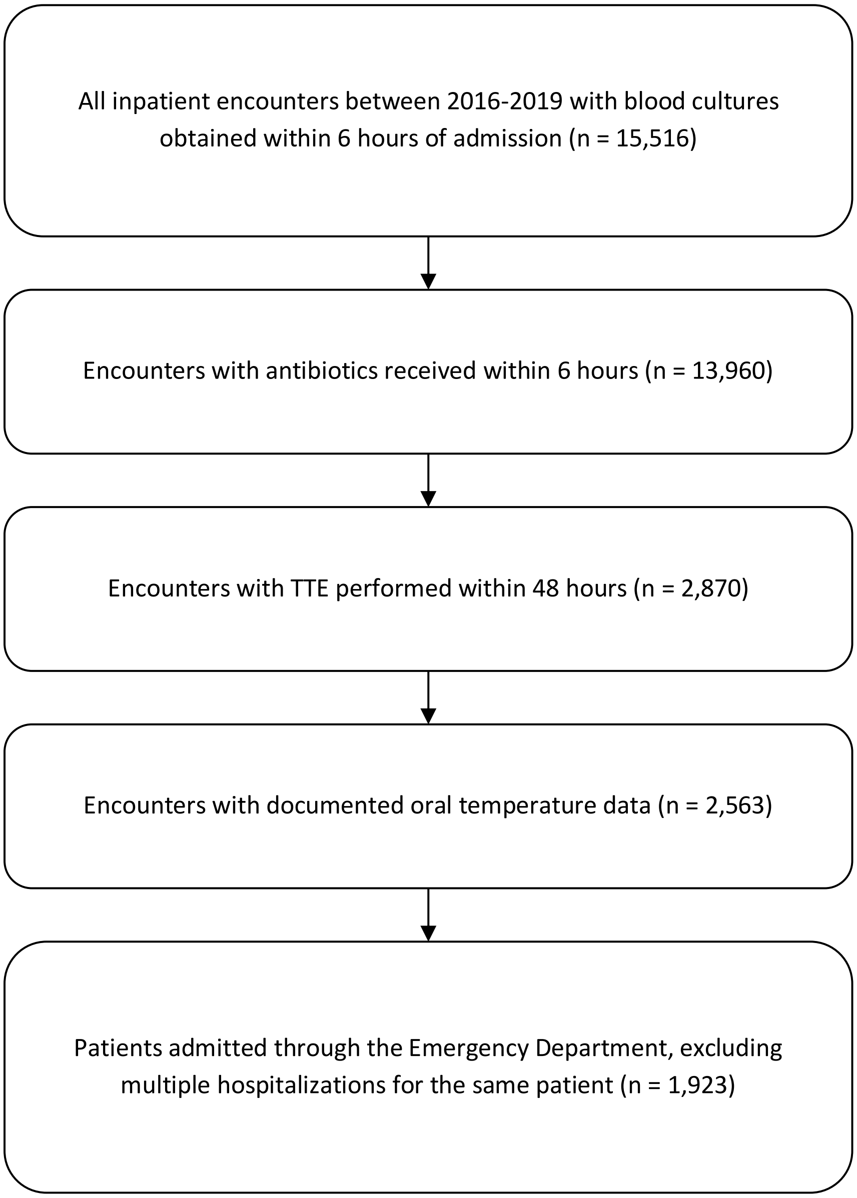

eFigure 2. Temperature trajectory subphenotypes in patients with suspected infection.
A cohort of 1,923 hospitalized septic patients were categorized into four previously validated temperature subphenotypes: hyperthermic, slow resolvers (n = 264, 14%), hyperthermic, fast resolvers (302, 16%), normothermics (903, 47%), and hypothermics (454, 24%). The figure illustrates the mean temperature measurements at each hour for each subphenotype. The shaded regions represent the 95% CI around the mean.

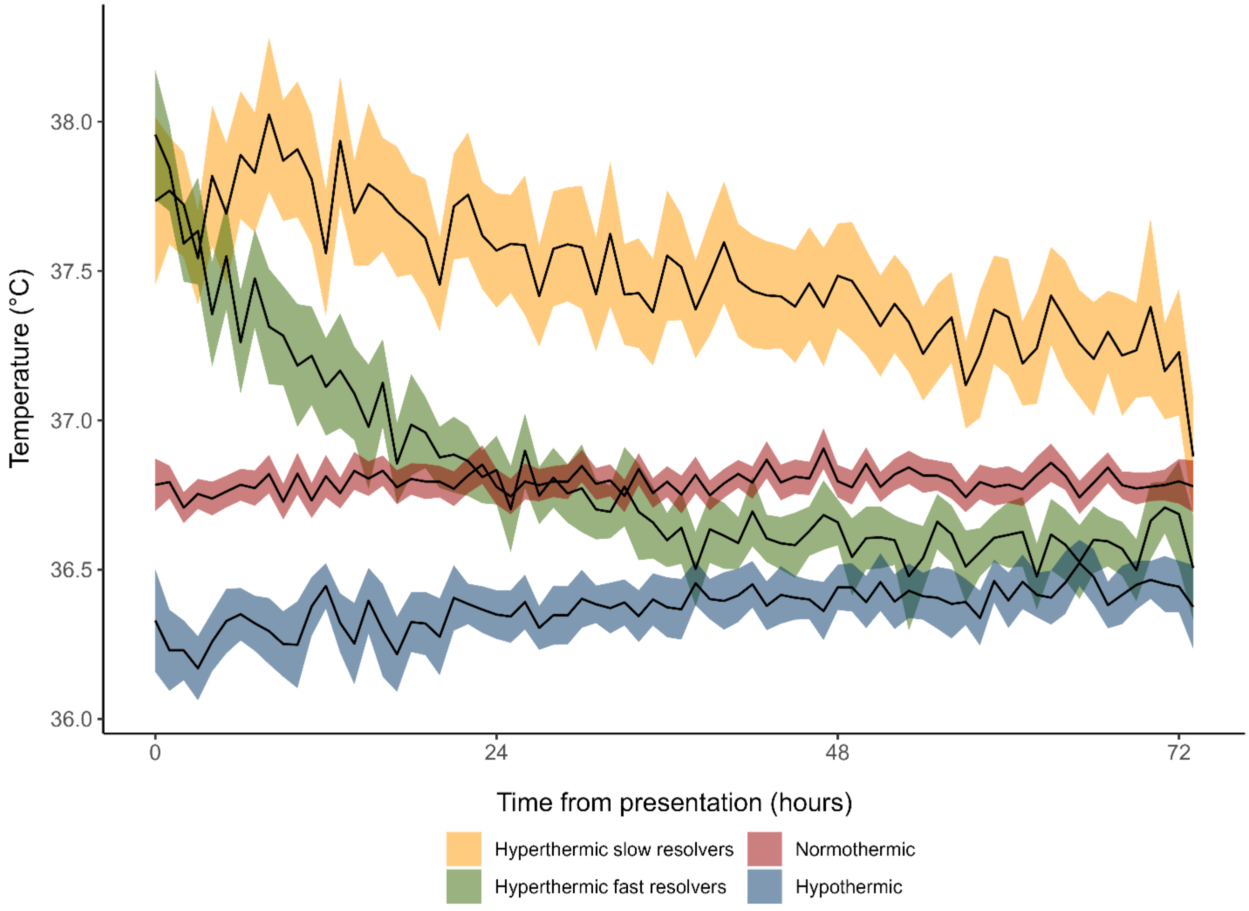

eFigure 3. Mean Admission Left Ventricular Ejection Fraction by Subphenotype.
The mean LVEF was significantly different between subphenotypes (p < *0.01*), with hyperthermic slow resolvers having a mean LVEF of 54.6% (12.5) and hypothermics having a mean LVEF of 47.1% (17.3). *The bar graph illustrates the mean and standard deviation of LVEF. Definition of abbreviations: HSR = hyperthermic, slow resolvers; HFR = hyperthermic, fast resolvers; NT = normothermic; HT = hypothermic.*

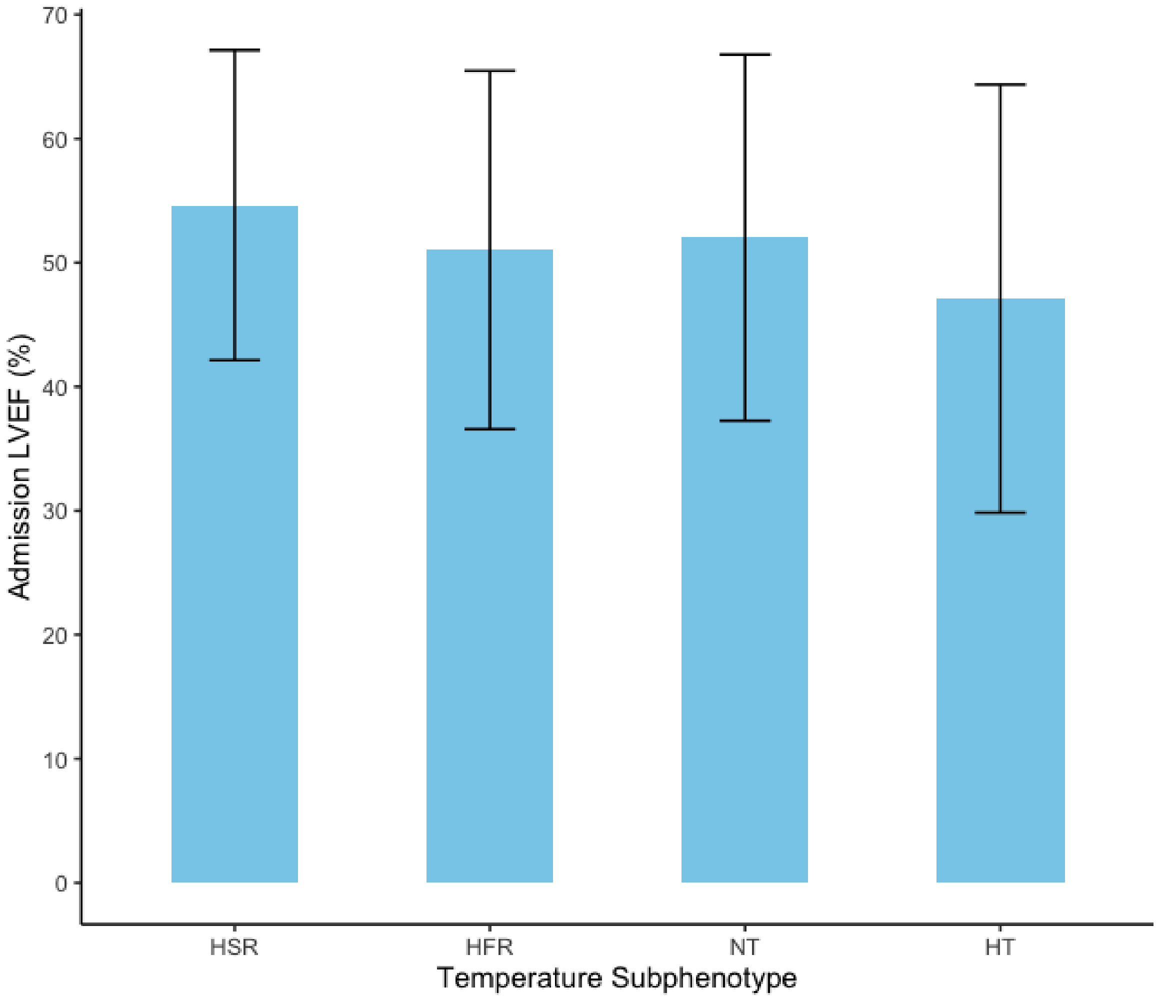

eFigure 4. Sensitivity Analysis with Normothermics as reference group.

We performed a sensitivity analysis using normothermics as the reference, and found that hypothermic subphenotype remains significantly associated with higher odds of reduced cardiac function (OR 1.58, 95% CI 1.24-2.01, p < *0.01*), while hyperthermic slow resolvers are associated with lower odds of reduced cardiac function (OR 0.60, 95% CI 0.43-0.83, p < *0.01*). Definition of abbreviations: HSR = hyperthermic, slow resolvers; HFR = hyperthermic, fast resolvers; NT = normothermic; HT = hypothermic.

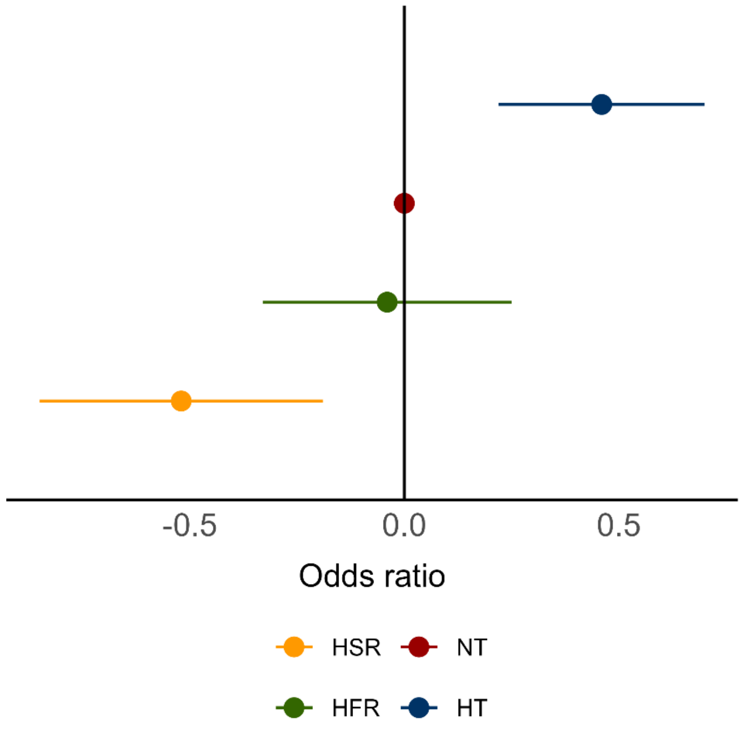

eFigure 5. Odds Ratio for LVEF ≤ 30% compared between temperature trajectory subphenotypes. Hypothermics had a significantly increased odds ratio of LVEF ≤ 30% (OR 1.14, 95% CI 1.08-1.21, p < *0.01*). Definition of abbreviations: HSR = hyperthermic, slow resolvers; HFR = hyperthermic, fast resolvers; NT = normothermic; HT = hypothermic.

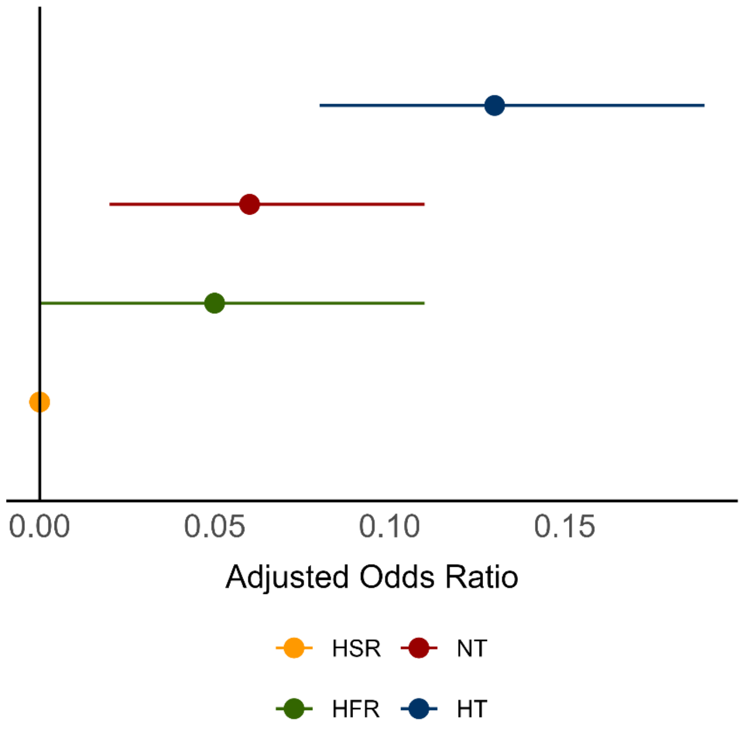

eFigure 6. Stratified Analysis by SOFA score.

We present stratified analyses by SOFA tertile, and found that across SOFA tertiles, hypothermics have significant association with reduced LVEF. With increasing severity of illness, more patients across all 4 subphenotypes are more likely to have cardiac dysfunction, and the strength of association with hypothermics and reduced LVEF decreases but remains significant. Definition of abbreviations: HSR = hyperthermic, slow resolvers; HFR = hyperthermic, fast resolvers; NT = normothermic; HT = hypothermic.

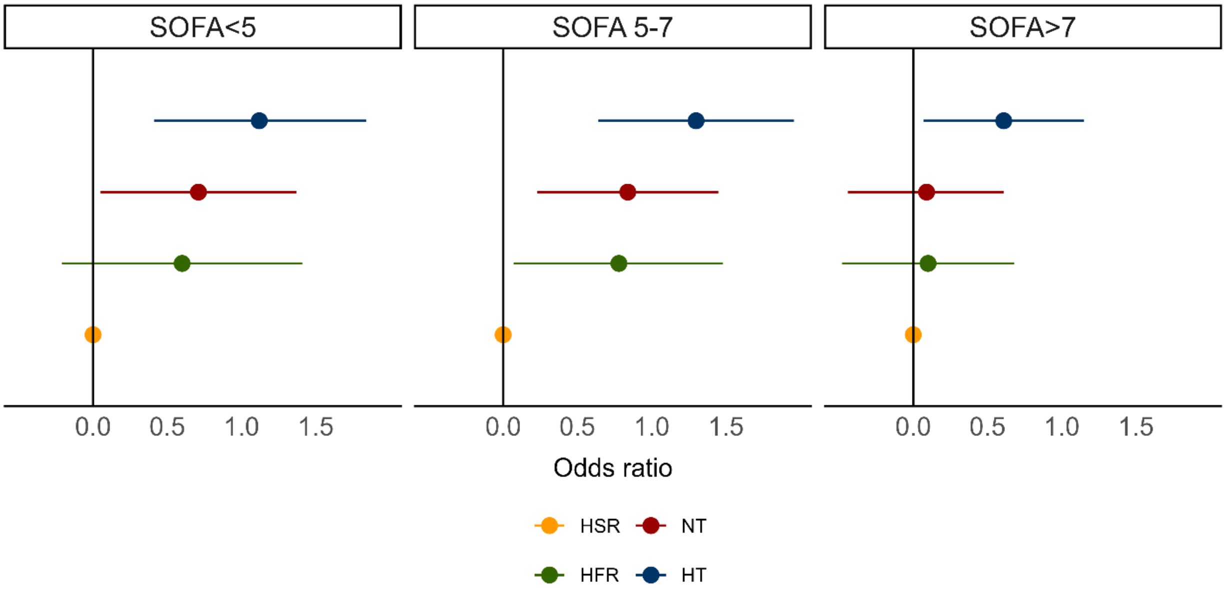

eFigure 7. Inotropes and Vasopressors compared between temperature trajectory subphenotypes. The hypothermic subphenotype had the highest percentage of patients receiving inotropes (dobutamine and milrinone), with over 3 times higher rates of use compared to the hyperthermic subphenotypes. Hypothermics also had the highest percentage receiving vasopressors, with over 20% of hypothermic patients having vasopressor use in the first day of hospitalization. Definition of abbreviations: HSR = hyperthermic, slow resolvers; HFR = hyperthermic, fast resolvers; NT = normothermic; HT = hypothermic.

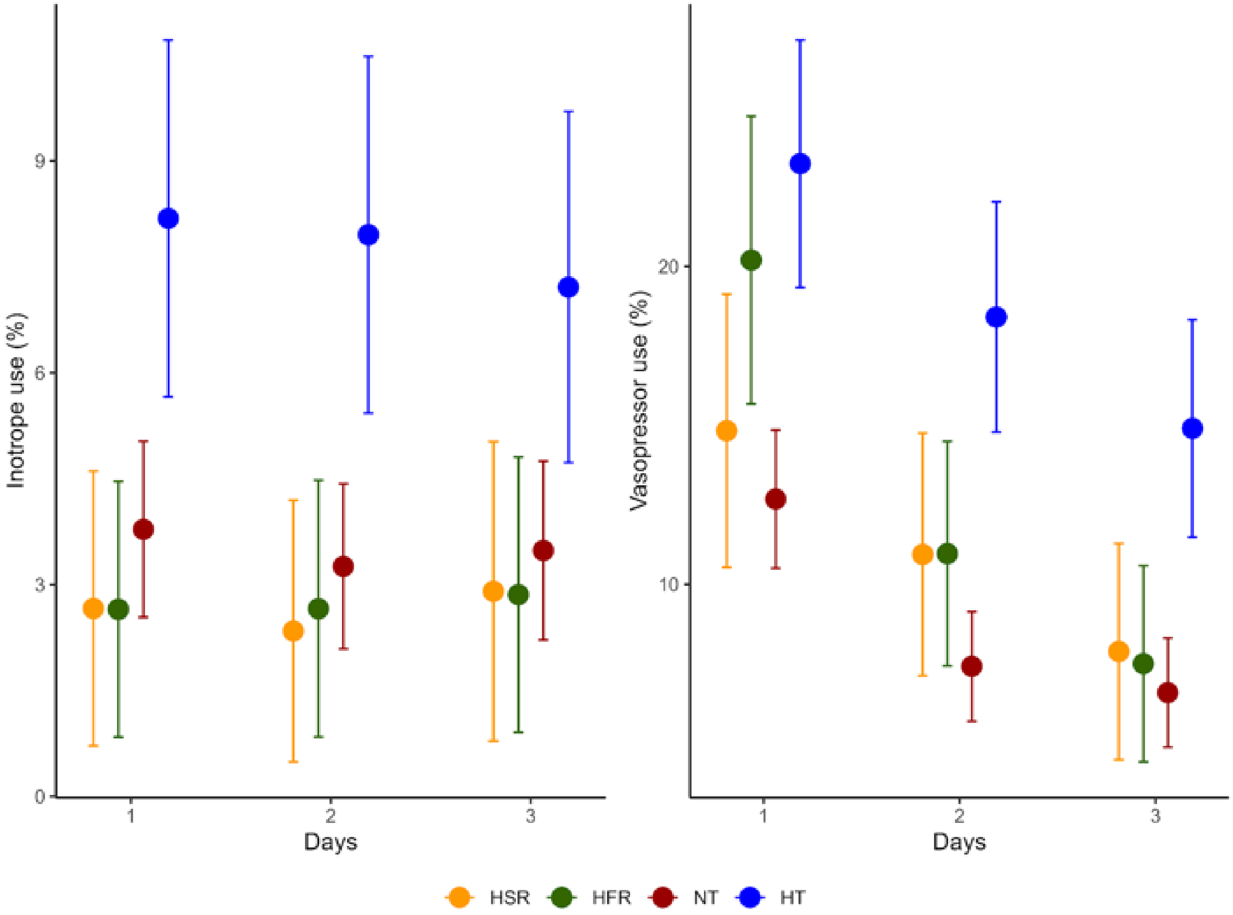

eFigure 8. Biomarker levels over time compared between temperature trajectory subphenotypes. Hypothermic patients had the higher lactate and creatinine levels, although the levels were not significantly different beyond day 1. Definition of abbreviations: HSR = hyperthermic, slow resolvers; HFR = hyperthermic, fast resolvers; NT = normothermic; HT = hypothermic.

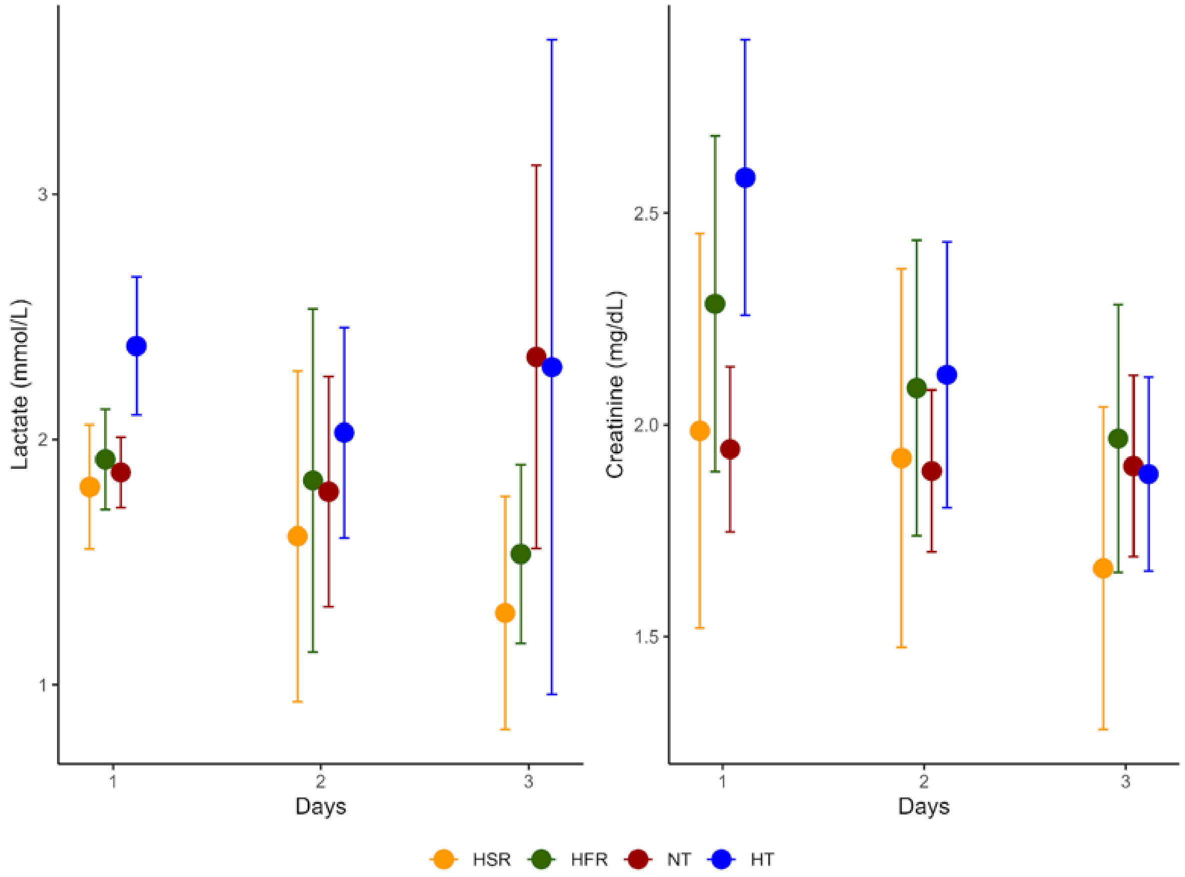

eFigure 9. 1-year Post Discharge LV dysfunction compared between temperature trajectory subphenotypes.

527 patients had repeat TTEs, with 71 HSR (representing 27% of HSR in the original study cohort), 86 HFR (28%), 227 NT (25%), and 143 HT (31%). On the post-discharge TTE, the association between hypothermic subphenotype and reduced LVEF persisted (OR 3.29, 95% CI 1.71-6.32, p < *0.01*), when adjusting for confounders and with hyperthermic slow resolvers as the reference.

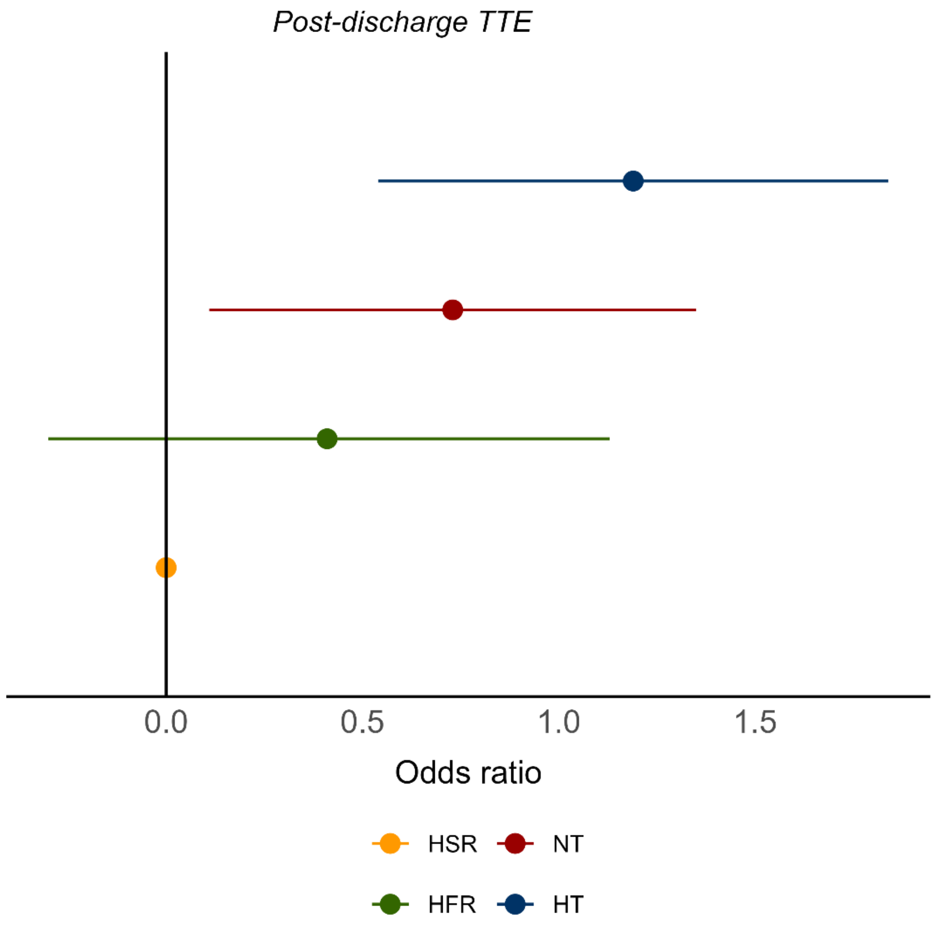

Supplement: Supplementary file 1 [file cc9-7-e1282-s001.pdf]
